# Supplementary material for: MetaQTL: a package of new computational methods for the meta-analysis of QTL mapping experiments
Source: BMC Bioinformatics. 2007 Feb 8;8:49. doi: 10.1186/1471-2105-8-49 (PMC1808479; doi:10.1186/1471-2105-8-49)
Supplement: Additional File 7 — MetaQTL Package : jar file and tutorial. This Zip archive contains both the MetaQTL JAR file and the files of the tutorial. [file 1471-2105-8-49-S7.zip › org.inra.metaqtl/doc/index-files/index-1.html]

A-Index


|  |  |  |
| --- | --- | --- |
| **Overview** \| Package \| Class \| Use \| **Tree** \| **Deprecated** \| **Index** \| **Help** | |  |
| PREV LETTER   **NEXT LETTER** | **FRAMES**    **NO FRAMES**     **All Classes** |  |


A B C D E F G H I K L M N O P Q R S T U V W X Y Z 

---


## **A**

**A2Xml** - Class in org.metaqtl.main: **A2Xml()** - Constructor for class org.metaqtl.main.A2Xml: **absolute()** - Method in interface org.thalia.bio.util.ILGroupPosition: **absolute()** - Method in class org.thalia.bio.util.LGroupPosition: **adapt(Chromosome[])** - Static method in class org.metaqtl.adapter.MetaQtlAnalysisAdapter: **adapt(EMResult)** - Static method in class org.metaqtl.adapter.QtlAdapter: From a result of a EM clustering returns the mixture components as an array of Qtl. **adapt(MetaQtlData)** - Static method in class org.metaqtl.adapter.QtlPartitionAdapter: Adapt the given `MetaQtlData` into a `QtlPartition` and returns it. **adapt(MetaQtlAnalysis, String, String, int)** - Static method in class org.metaqtl.adapter.QtlPartitionAdapter: Adapt the `MetaQtlAnalysis` `analysis` for a given value of the number of clusters `k` and a given trait name `code`. **adapt(MetaQtlResult, Qtl[], int)** - Static method in class org.metaqtl.adapter.QtlPartitionAdapter: **addAllele(IBioAllele)** - Method in class org.thalia.bio.entity.AlleleContainer: **addAllele(IBioAllele)** - Method in interface org.thalia.bio.IBioLocus: **addAllMap(IBioGenome[])** - Method in class org.metaqtl.MapCluster: **addBooleanOption(char, String)** - Method in class org.metaqtl.main.CmdLineParser: Convenience method for adding a boolean option. **addBooleanOption(String)** - Method in class org.metaqtl.main.CmdLineParser: Convenience method for adding a boolean option. **addChild(TreeNode)** - Method in class org.metaqtl.TreeNode: Add a child to the node. **addChromosome(IBioLGroup, IBioCross, int, int, boolean, boolean)** - Method in class org.metaqtl.ChromCluster: Add a chromosome to the cluster **addChromosome(IBioLGroup, IBioCross, int, int, boolean, boolean)** - Method in class org.metaqtl.MapCluster: Add a chromosome to the map cluster framework. **addDoubleOption(char, String)** - Method in class org.metaqtl.main.CmdLineParser: Convenience method for adding a double option. **addDoubleOption(String)** - Method in class org.metaqtl.main.CmdLineParser: Convenience method for adding a double option. **addEdge(Chromosome, Chromosome, CMarkerSequence)** - Method in class org.metaqtl.ChromCluster.ClusterGraph: **addGroup(IBioLGroup)** - Method in class org.thalia.bio.entity.GroupContainer: **addGroup(IBioLGroup)** - Method in interface org.thalia.bio.IBioGenome: **addIndividual(IBioIndividual)** - Method in class org.thalia.bio.entity.IndividualContainer: **addIndividual(IBioIndividual)** - Method in interface org.thalia.bio.IBioPopulation: **addIntegerOption(char, String)** - Method in class org.metaqtl.main.CmdLineParser: Convenience method for adding an integer option. **addIntegerOption(String)** - Method in class org.metaqtl.main.CmdLineParser: Convenience method for adding an integer option. **additive** - Variable in class org.metaqtl.Qtl.QTLStats: The additive effect of the QTL. **addLocus(IBioLocus)** - Method in class org.thalia.bio.entity.LocusContainer: **addLocus(IBioLocus)** - Method in interface org.thalia.bio.IBioLGroup: **addLongOption(char, String)** - Method in class org.metaqtl.main.CmdLineParser: Convenience method for adding a long integer option. **addLongOption(String)** - Method in class org.metaqtl.main.CmdLineParser: Convenience method for adding a long integer option. **addMap(IBioGenome, boolean)** - Method in class org.metaqtl.MapCluster: Add a map to the cluster. **addOption(CmdLineParser.Option)** - Method in class org.metaqtl.main.CmdLineParser: Add the specified Option to the list of accepted options **addQtl(Graphics2D, QtlUnit)** - Method in class org.metaqtl.graph.QtlLayerBuffer: Try to add a qtl unit to the configuration. **addStringOption(char, String)** - Method in class org.metaqtl.main.CmdLineParser: Convenience method for adding a string option. **addStringOption(String)** - Method in class org.metaqtl.main.CmdLineParser: Convenience method for adding a string option. **addTerm(String, String)** - Method in class org.metaqtl.MetaDico: **adjTable** - Variable in class org.metaqtl.ChromCluster.ClusterGraph: **AIC** - Static variable in class org.metaqtl.EMCriteria: **aic** - Variable in class org.metaqtl.EMCriteria: **AIC3** - Static variable in class org.metaqtl.EMCriteria: **aic3** - Variable in class org.metaqtl.EMCriteria: **AIC3\_NAME** - Static variable in class org.metaqtl.EMCriteria: **AIC\_NAME** - Static variable in class org.metaqtl.EMCriteria: **AICc** - Static variable in class org.metaqtl.EMCriteria: **aicc** - Variable in class org.metaqtl.EMCriteria: **AICc\_NAME** - Static variable in class org.metaqtl.EMCriteria: **ALIGN\_FIRST\_COMMON** - Static variable in class org.metaqtl.graph.ChromLayer: **ALIGN\_FIRST\_MARKER** - Static variable in class org.metaqtl.graph.ChromLayer: **alignChromLayers(ChromLayer, ChromLayer, int)** - Static method in class org.metaqtl.graph.ChromLayer: **ALLELE** - Static variable in interface org.thalia.bio.IBioEntityTypes: The integer code for Allele entity **AlleleContainer** - Class in org.thalia.bio.entity: Class Description Here **AlleleContainer()** - Constructor for class org.thalia.bio.entity.AlleleContainer: **AlleleContainer(String, IBioEntity)** - Constructor for class org.thalia.bio.entity.AlleleContainer: **alleles()** - Method in class org.thalia.bio.entity.AlleleContainer: **alleles()** - Method in interface org.thalia.bio.IBioLocus: **AnchoredElement** - Class in org.metaqtl.graph: **AnchoredElement()** - Constructor for class org.metaqtl.graph.AnchoredElement: **areMarkersAroundQTL(int, int, int)** - Method in class org.metaqtl.Chromosome: Returns true if the QTL position is inside the marker interval which markers indices are `mrkIdxL` and `mrkIdxR`. **attach(Object)** - Method in class org.metaqtl.graph.ChromLayer: **attach(Object)** - Method in class org.metaqtl.graph.Layer: Attach the given `object` to the layer. **attach(Object)** - Method in class org.metaqtl.graph.QtlLayer: **attach(Object)** - Method in class org.metaqtl.graph.QtlPartitionLayer: **attach(Object)** - Method in class org.metaqtl.graph.QtlTreeLayer: **attachQtl(Qtl[])** - Method in class org.metaqtl.Chromosome: **attachQTL(IBioLocus[])** - Method in class org.metaqtl.Chromosome: Add **AWE** - Static variable in class org.metaqtl.EMCriteria: **awe** - Variable in class org.metaqtl.EMCriteria: **AWE\_NAME** - Static variable in class org.metaqtl.EMCriteria

---


|  |  |  |
| --- | --- | --- |
| **Overview** \| Package \| Class \| Use \| **Tree** \| **Deprecated** \| **Index** \| **Help** | |  |
| PREV LETTER   **NEXT LETTER** | **FRAMES**    **NO FRAMES**     **All Classes** |  |


A B C D E F G H I K L M N O P Q R S T U V W X Y Z 

---
